# Regulation of Second Basal Internode Characteristics by Nitrogen Fertilizer Enhances Lodging Resistance and Yield in Winter Wheat (*Triticum aestivum* L.)

**DOI:** 10.3390/plants15071089

**Published:** 2026-04-02

**Authors:** Chong Shang, Qianwen Li, Weiwei Duan, Jinkao Guo, Baoyuan Zhou, Jiayu Ma, Li Wang, Xuejing Liu, Wenchao Zhen

**Affiliations:** 1 College of Agronomy, Hebei Agricultural University, Baoding 071001, China; 13313120751@163.com (C.S.); 15369856835@163.com (Q.L.); duanweiwei@hebau.edu.cn (W.D.); 13673148166@163.com (J.M.); 2 Key Laboratory of North China Water-Saving Agriculture, Ministry of Agriculture and Rural Affairs, Baoding 071001, China; 3 State Key Laboratory of North China Crop Improvement and Regulation, Baoding 071001, China; sjzgjk@163.com; 4 Wheat Research Center, Shijiazhuang Academy of Agriculture and Forestry Sciences, Shijiazhuang 050041, China; 5 Institute of Crop Science, Chinese Academy of Agricultural Sciences, Beijing 100081, China; zhoubaoyuan@caas.cn; 6 State Key Laboratory of Crop Gene Resources and Breeding, Beijing 100081, China; 7 School of Landscape and Ecological Engineering, Hebei University of Engineering, Handan 056038, China; 8 College of Mining Engineering, North China University of Science and Technology, Tangshan 063009, China; liuxj@ncst.edu.cn

**Keywords:** winter wheat, nitrogen basal-to-topdressing ratio, second basal internode (I2), culm lodging resistance index (CLRI), milk stage, yield

## Abstract

In the North China Plain (NCP), wind and rain during the grain-filling period of winter wheat can cause lodging. The second basal internode (I2), a key load-bearing structure, plays a central role in yield stability. This study, under a constant nitrogen (N) application rate of 270 kg ha^−1^, aimed to clarify how nitrogen basal-to-topdressing ratios regulate I2 characteristics to balance lodging resistance and yield increase. Field experiments were conducted across two seasons with three cultivars and three nitrogen split ratios (5:5, CK; 3:7, N1; and 7:3, N2). Dynamic measurements of I2 mechanical properties, morphology, anatomy, and composition were taken, and structural equation modeling (SEM) was used for analysis. Results showed that the culm lodging resistance index (CLRI) decreased by 41.8% from flowering to milk stage under all treatments, with CLRI at the milk stage of lodging treatments between 0.11 and 0.15. SEM supported a composition–structure–lodging resistance–yield chain, with CLRI as the key mediator. The N1 treatment significantly improved CLRI at all stages and increased yield by 12.2% compared to CK, making it a recommended nitrogen strategy for improving both yield and lodging resistance. These findings provide agronomically applicable nitrogen management guidelines for high-yield winter wheat systems.

## 1. Introduction

Wheat (*Triticum aestivum* L.) is one of the world’s major staple crops, providing dietary calories for ~40% of the global population [1]. The North China Plain (NCP), a key winter wheat production region, plays a pivotal role in safeguarding regional food security [2,3]. However, with the rapid replacement of high-yielding cultivars and the continual increase in grain yield, the risk of lodging has intensified. In recent years, lodging has affected approximately 5–10% of the wheat area in the NCP annually, exceeding 20% in severe years [4]. Field survey data indicate that slight lodging typically reduces yield by 10–20%, whereas severe lodging can cause yield losses of up to 80% and, in extreme cases, complete crop failure [5,6]. Lodging results from the combined effects of genetic background, agronomic management, and meteorological stress, among which nitrogen management and spring canopy regulation are particularly critical [7,8]. Rational nitrogen management can effectively alleviate the trade-off between individual plant growth and population performance and improve canopy structure, whereas excessive or improperly timed topdressing often weakens culm quality and deteriorates stand architecture [8,9]. Therefore, developing and implementing effective nitrogen (N) management strategies is a prerequisite for simultaneously achieving high yield and enhanced lodging resistance.

N management mainly comprises two components—an appropriate total N application rate and an optimized basal-to-topdressing split—and is a key pathway for improving crop productivity [10,11]. In recent years, many studies have largely converged on the general effects of total N input on wheat performance, showing that excessive N supply tends to promote luxuriant vegetative growth of stems and leaves, reduce post-anthesis translocation of carbohydrates to the grain, and is often accompanied by elongated basal internodes, thinner culm walls, and declines in internode filling degree (TFD) and mechanical strength [8,12,13,14]. In contrast, an adequate and balanced fertilizer supply can improve nutrient translocation from stems and leaves to the grain and thereby increase yield [15]. Beyond total N rate, split N application—particularly strategies that shift N supply from early to later growth stages—has been reported to enhance lignin accumulation in wheat culms, increase breaking strength (BS), and ultimately improve lodging resistance [15,16]. However, other studies have suggested that sufficient basal fertilization combined with split applications can simultaneously increase grain yield and culm strength [17,18,19,20]. Overall, there is broad agreement that split N application is beneficial for strengthening culms, whereas conclusions regarding the optimal basal-to-topdressing N ratio remain inconsistent across experimental conditions and therefore warrant further investigation.

Under production conditions in NCP, windy and rainy weather frequently occurs during the mid-to-late grain-filling period. At this time, a rapid decline in culm mechanical strength coincides with increasing structural loading, leading to a sharp escalation in lodging risk [21,22]. When the total N rate is fixed, optimizing the proportion of spring topdressing represents an effective lever to enhance culm strength during the late growth stages [15]. From a biological perspective, the basal internodes are among the most critical load-bearing and stabilizing components of the culm, with the second basal internode (I2) playing a particularly important role in supporting the spike and upper canopy and in resisting bending and breakage [5,23]. However, culm strength is typically an emergent property resulting from coordination across hierarchical traits: morphological architecture determines the basic geometry and material distribution; anatomical structure defines the tissue-level load-bearing framework; and culm composition governs cell-wall reinforcement and structural quality [24,25,26]. These attributes may operate in a cascade. At the compositional level, lignin accumulation in basal internodes and its monomer composition are closely associated with stem breaking strength [27,28]; at the structural level, anatomical indicators have been shown to be significantly related to lodging resistance, and these tissue-structural features are responsive to nitrogen management [29]. Collectively, this evidence provides support for a chain relationship in which composition shapes structure and structure determines mechanical function.

Overall, although substantial progress has been made, most existing studies still primarily focus on the total N rate or evaluate only a limited set of lodging-related traits at a single growth stage [11,12,16], and therefore provide limited insight into how N application timing reshapes the dynamic, coordinated multi-scale reinforcement process of the key load-bearing internode across morphology–anatomy–composition traits. More importantly, previous work generally lacks systematic integration and mechanistic pathway quantification of the “composition–structure–function” cascade within a single experimental system [27,28,29], which limits the development of interpretable and transferable mechanistic evidence for management optimization. Accordingly, it is necessary to build a dynamic, multi-scale evidence chain centered on I2 within the lodging-sensitive window and to integrate multi-scale traits within a unified mechanistic framework, thereby systematically elucidating the intrinsic mechanisms that link lodging resistance with yield formation.

**Figure 1:**
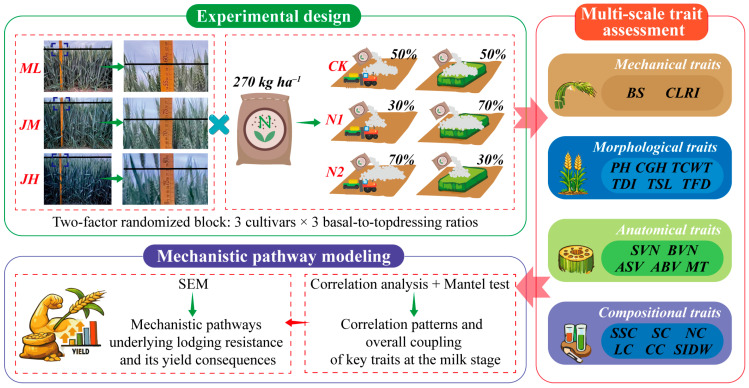
Conceptual and analytical workflow of this study.

Accordingly, we selected high-yielding cultivars differing in lodging resistance and conducted multi-year field experiments under a constant total N rate with contrasting basal-to-topdressing N split ratios. Lodging-related traits were systematically monitored at three key developmental stages, and the overall workflow is illustrated in Figure 1. The specific objectives were to: (i) quantify the interactive effects of N split ratio and cultivar on yield formation and lodging risk, and to identify an optimal N-splitting strategy that balances high yield with improved lodging resistance; (ii) clarify how the decline in I2 mechanical performance is coupled with changes in morphological architecture, anatomical structure, and culm compositional traits, thereby explaining lodging-resistance differences among N-splitting strategies; and (iii) integrate multi-scale traits to delineate the mechanistic pathways underpinning lodging resistance and their yield consequences, and to identify adjustable bottlenecks and core trait modules that can be targeted for management optimization. Collectively, these outcomes provide an operational basis for N management in high-yielding winter wheat systems of the NCP, enabling yield stabilization while reducing lodging risk, and offer guidance on trait-improvement priorities for lodging-resistance breeding.

## 2. Results

### 2.1. Effects of the Interaction Between Nitrogen Basal-to-Topdressing Ratio and Cultivar on Yield Formation

**Figure 2:**
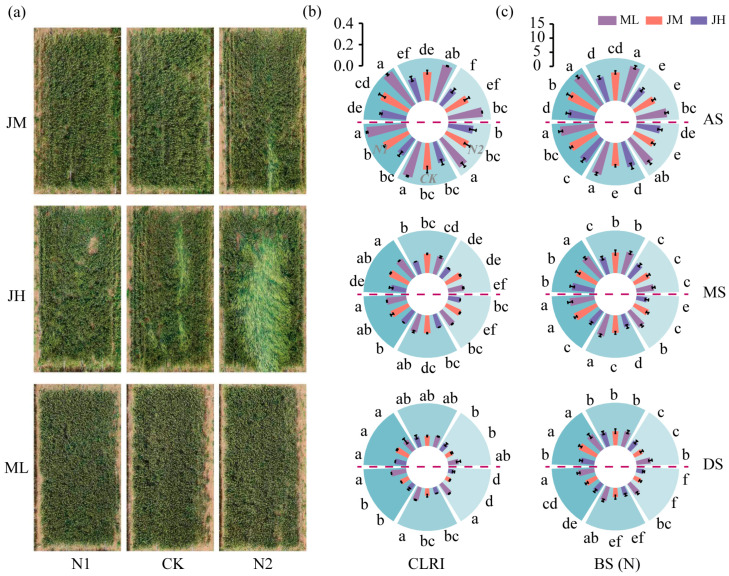
Effects of nitrogen fertilization on lodging resistance in different winter wheat cultivars. (**a**) Aerial photograph of all treatments at the time of lodging occurrence in the 2022–2023 growing season. (**b**) Culm lodging resistance index of the second internode (CLRI) and (**c**) breaking strength of the second internode (BS). Data above the horizontal dashed line represent the 2021–2022 growing season, and those below represent the 2022–2023 growing season. AS, anthesis; MS, milk stage; DS, dough stage. Different lowercase letters indicate significant differences among treatments within the same season at *p* < 0.05 (*n* = 3). Vertical bars above mean values indicate standard deviations.

This study showed that grain yield and its components of winter wheat were significantly affected by year (Y), cultivar (V), and the basal-to-topdressing N ratio, with generally significant interaction effects; only the three-way interaction for grain number per spike was not significant (Table 1). Specifically, the mean yield in the 2022–2023 growing season was 18.4% lower than that in 2021–2022, which was associated with lodging occurring around the milk stage in that season (Figure 2a). Relative to the reference treatment (CK), N1 significantly increased grain number per spike (by 3.8%), thousand-grain weight (by 4.0%), and yield (by 12.2%) across cultivars, whereas spike number was increased significantly only in ML (by 8.6%). In contrast, N2 significantly reduced all yield components and yield across cultivars. Across the two seasons, ML consistently achieved the highest yield, exceeding JM and JH by 5.1% and 12.2%, respectively.

### 2.2. Association Between the Decline in I2 Mechanical Traits and Lodging Occurrence at the Milk Stage

No lodging was observed in any treatment during the 2021–2022 growing season. In contrast, lodging occurred to varying degrees in JH and JM in the 2022–2023 season (Figure 2a). Under N2, the lodging grade of JH and JM was III and I, respectively, with lodging rates of 74.86% and 2.27%. Under CK, JH exhibited grade II lodging, with a lodging rate of 14.29%. All lodging events occurred at the milk stage and coincided with wind–rain episodes.

Both mechanical traits of I2, i.e., culm lodging resistance index (CLRI) and BS, decreased progressively with crop development (Figure 2b,c). Notably, CLRI declined sharply by 41.8% from anthesis to the milk stage, which temporally matched the lodging-prone period. Across nitrogen split treatments, N1 generally resulted in higher CLRI and BS, whereas N2 significantly reduced both traits in most cases. Across cultivars, ML consistently maintained the highest CLRI and BS across growth stages, averaging 27.1% and 30.5% higher than JM, and 53.3% and 42.2% higher than JH, respectively. Importantly, the lodging treatments (JHCK, JHN2, and JMN2) exhibited markedly lower CLRI at the milk stage (0.11–0.15).

### 2.3. Responses of I2 Morphological Traits and Their Links to Culm-Strength Formation

**Figure 3:**
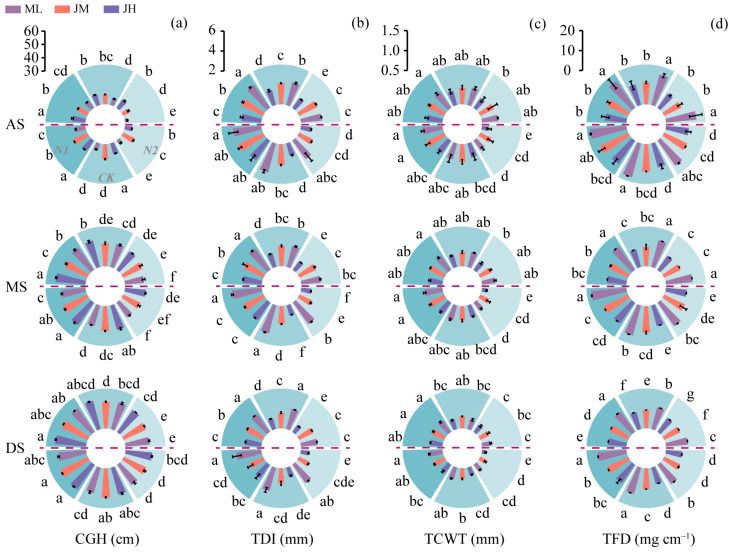
Effects of nitrogen fertilization on (**a**) center of gravity height (CGH), (**b**) stem diameter of the second internode (TDI), (**c**) culm wall thickness of the second internode (TCWT), and (**d**) culm filling degree of the second internode (TFD) in different winter wheat cultivars. Data above the horizontal dashed line represent the 2021–2022 growing season, and those below represent the 2022–2023 growing season. AS, anthesis; MS, milk stage; DS, dough stage. Different lowercase letters indicate significant differences among treatments within the same season at *p* < 0.05 (*n* = 3). Vertical bars above mean values indicate standard deviations.

**Figure A1:**
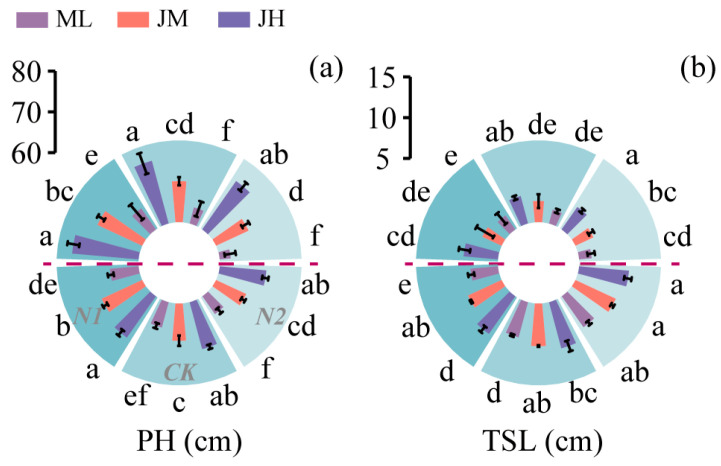
Effects of nitrogen fertilization on (**a**) plant height (PH) and (**b**) second basal internode (I2) length (TSL) at anthesis in different winter wheat cultivars. Data above the horizontal dashed line represent the 2021–2022 growing season, and data below represent the 2022–2023 growing season. Different lowercase letters indicate significant differences among treatments within the same year at *p* < 0.05 (*n* = 3). Vertical bars above mean values indicate standard deviations.

Morphological traits differed markedly among cultivars, with plant height (PH) being significantly higher in JH than in ML and JM by 11.57% and 4.01% on average, respectively (Figure A1a). Across most cases, N1 increased PH and elevated the center of gravity height (CGH) (Figure 3a), while concurrently shortening I2 (Figure A1b). The I2 diameter (TDI), culm wall thickness (TCWT), and TFD exhibited an overall decline as development progressed, decreasing by 12.7%, 15.3%, and 22.0% on average (Figure 3b–d). Overall, N1 sustained greater I2 structural robustness, with TDI, TCWT, and TFD being 18.7%, 17.8%, and 18.8% higher than under N2 (and 10.8%, 5.7%, and 9.1% higher than under CK), respectively. At the cultivar level, ML showed the strongest advantage in I2 robustness: TDI and TFD were on average 16.1% and 30.5% higher than in JM and 32.3% and 42.2% higher than in JH, respectively, and TCWT was 10.3% higher than in JH.

### 2.4. Anatomical Basis Underpinning Culm Strength Formation of I2

**Figure 4:**
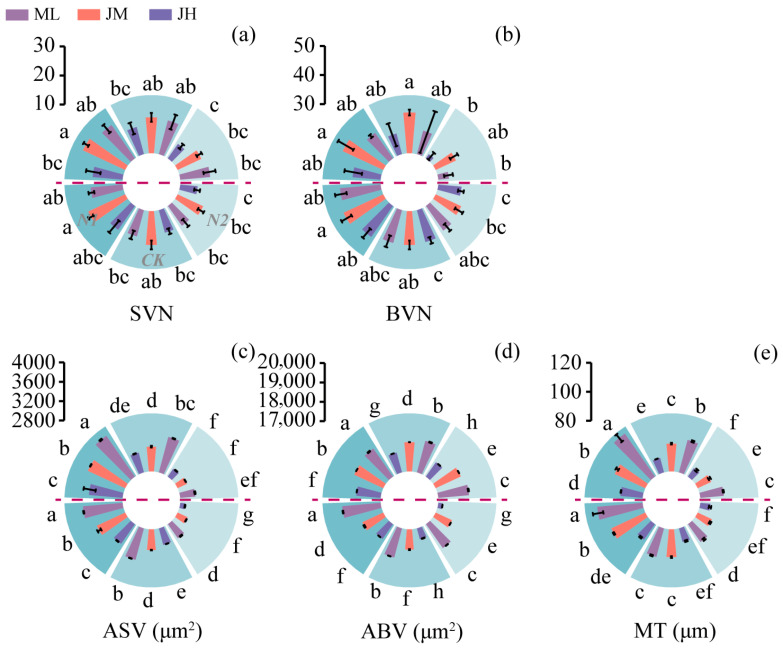
Effects of nitrogen fertilization on anatomical traits of the I2 (second internode) in different winter wheat cultivars. (**a**–**e**) represent the number of small vascular bundles (SVNs), number of large vascular bundles (BVNs), area of small vascular bundles (ASVs), area of large vascular bundles (ABVs), and mechanical tissue thickness (MT) at the milk stage, respectively. Data above the horizontal dashed line represent the 2021–2022 growing season, and those below represent the 2022–2023 growing season. Different lowercase letters indicate significant differences among treatments within the same season at *p* < 0.05 (*n* = 3). Vertical bars above mean values indicate standard deviations.

Anatomical traits indicated that N1 generally promoted vascular bundle development and tissue reinforcement: the numbers of small and large vascular bundles (SVN and BVN) were, on average, 23.5% and 16.6% higher under N1 than under N2 (Figure 4a,b). The areas of small and large vascular bundles (ASV and ABV) and mechanical tissue thickness (MT) were, on average, 16.4%, 4.0%, and 14.4% higher under N1 than under N2, respectively (corresponding to increases of 7.6%, 2.4%, and 7.4% relative to CK; Figure 4c–e). At the cultivar level, ML exhibited higher ABV and MT than JH by 9.7% and 14.7% on average, with ASV also increasing by 8.5%; JM showed the highest SVN and BVN, exceeding those of JH by 21.0% and 11.5%, respectively, yet remained inferior to ML in MT.

### 2.5. Relationships Between I2 Compositional Traits and Culm Strength Formation

**Figure 5:**
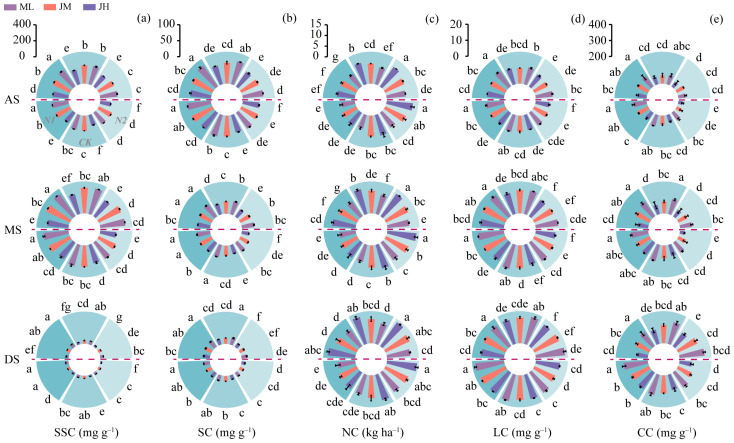
Effects of nitrogen fertilization on the second basal internode (I2) (**a**) soluble sugar content (SSC), (**b**) starch content (SC), (**c**) nitrogen content (NC), (**d**) lignin content (LC), and (**e**) cellulose content (CC) in different winter wheat cultivars. Data above the horizontal dashed line represent the 2021–2022 growing season, and those below represent the 2022–2023 growing season. AS, anthesis; MS, milk stage; DS, dough stage. Different lowercase letters indicate significant differences among treatments within the same season at *p* < 0.05 (*n* = 3). Vertical bars above mean values indicate standard deviations.

**Figure A2:**
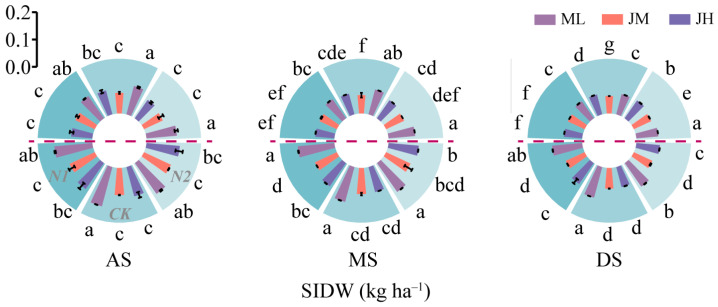
Effects of nitrogen fertilization on the second basal internode (I2) dry weight (SIDW) in different winter wheat cultivars. Data above the horizontal dashed line represent the 2021–2022 growing season, and those below represent the 2022–2023 growing season. AS, anthesis; MS, milk stage; DS, dough stage. Different lowercase letters indicate significant differences among treatments within the same season at *p* < 0.05 (*n* = 3). Vertical bars above mean values indicate standard deviations.

The compositional traits of I2 showed that soluble sugar content (SSC) generally increased after anthesis and peaked at the milk stage before declining (Figure 5a), whereas starch content (SC) decreased progressively with crop development (Figure 5b). In contrast, lignin content (LC) and cellulose content (CC) accumulated steadily over time (Figure 5d,e), indicating that the period around the milk stage represents a critical window for carbon pool reallocation and cell-wall reinforcement. Across years, SSC was consistently lower in 2022–2023 than in 2021–2022 at all growth stages (by 13.6–21.2%), which aligns with the overall pattern of reduced I2 strength in the lodging year. At the nitrogen split level, N1 generally maintained higher SSC and SC and promoted the accumulation of LC and CC, whereas N2 exhibited greater I2 nitrogen content (NC), averaging 30.2% and 11.9% higher than N1 and CK, respectively (Figure 5c). Among cultivars, ML tended to show higher SSC, SC, LC, and CC than JM and JH, while JH exhibited higher NC. Notably, SIDW declined overall by 22.1% with advancing development (Figure A2), yet was higher under N2 than under N1 and CK (by 4.3% on average), suggesting that dry matter accumulation per se cannot substitute for compositional quality in explaining culm strength formation.

### 2.6. Correlation Patterns and Overall Coupling of Key Traits at the Milk Stage

**Figure 6:**
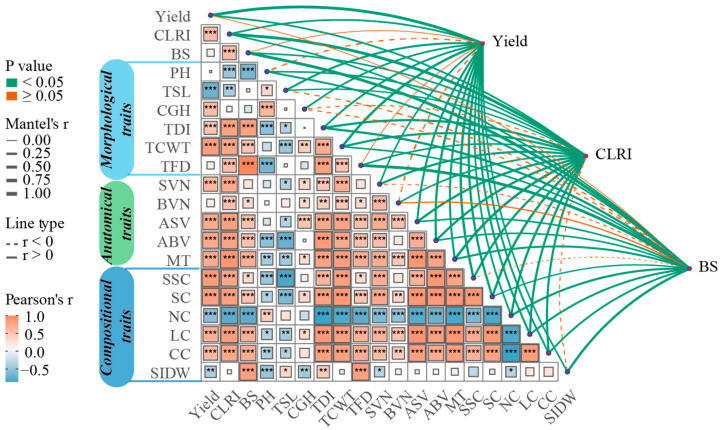
Pearson correlation matrix of candidate traits at the milk stage (MS) and their Mantel-based coupling with grain yield, the culm lodging resistance index (CLRI), and bending strength (BS) (*n* = 54). Significance is indicated as *p* < 0.05 (*), *p* < 0.01 (**), and *p* < 0.001 (***).

Correlation analysis showed that yield was significantly and positively correlated with CLRI (r = 0.54), whereas it was not significantly correlated with BS (Figure 6). This distinction is reasonable because BS is a purely mechanical strength indicator of the internode, whereas CLRI is a comprehensive lodging-resistance index; consequently, their correlation patterns with yield differ. This also indicates that a single mechanical-strength metric alone is insufficient to account for yield variation. CLRI was significantly and positively associated with TDI, TCWT, and most anatomical traits and I2 compositional traits, but was significantly negatively associated with PH, TSL, and CGH, which collectively reflect a taller or elongated morphology. These relationships suggest that lodging resistance at the milk stage is jointly underpinned by I2 structural reinforcement and enhanced cell-wall composition. Notably, CGH showed a more consistent positive trend with yield, yet its correlation with CLRI was not significant. Mantel tests based on Euclidean distance matrices further corroborated these overall patterns, revealing significant global associations between the candidate trait set and yield, CLRI, and BS (all *p* < 0.05). Among TDI, TCWT, the anatomical trait MT, and compositional traits (SSC, SC, LC, and NC) contributed more prominently to the overall coupling with these three response variables.

### 2.7. Mechanistic Pathways Underlying Lodging Resistance and Its Yield Consequences

**Figure 7:**
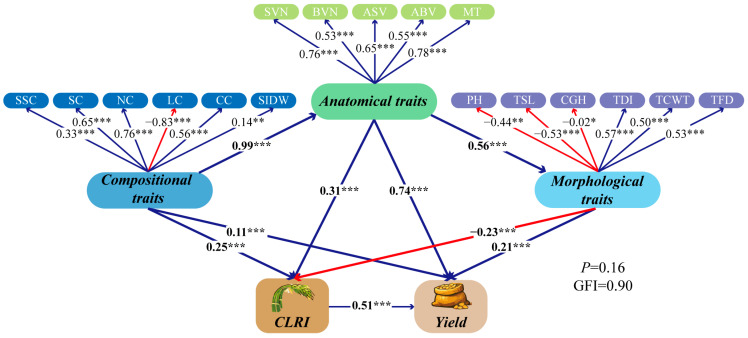
Mechanistic pathways underlying lodging resistance at the milk stage (MS) and their effects on grain yield (*n* = 54). Values adjacent to arrows are standardized path coefficients (blue, positive; red, negative). Path significance is indicated as *p* < 0.05 (*), *p* < 0.01 (**), and *p* < 0.001 (***).

The structural equation model (SEM) exhibited an acceptable overall fit (χ^2^ test *p* = 0.16; GFI = 0.90) and, within the biological-hypothesis framework, resolved a transmission chain of “composition—structure—lodging resistance—yield” (Figure 7). I2 composition exerted a very strong positive effect on anatomical traits (standardized path coefficient = 0.99 ***), and anatomical traits further promoted morphological traits (0.56 ***). Anatomical traits had significant direct effects on both CLRI and yield (0.31 *** and 0.74 ***, respectively). In contrast, morphological traits showed a negative direct effect on CLRI (−0.23 ***) but a positive direct effect on yield (0.21 ***). In addition, I2 composition retained smaller yet significant direct effects on CLRI and yield (0.25 *** and 0.11 ***, respectively). Importantly, CLRI acted as a key mediator that significantly increased yield (0.51 ***), thereby translating structural reinforcement into measurable yield gains.

## 3. Discussion

### 3.1. Recommended Basal-to-Topdressing N Ratio Balancing Grain Yield and Lodging Resistance

In high-yielding systems, yield-enhancing practices often trade off against lodging risk [30]; thus, the basal-to-topdressing N ratio essentially serves as a tuning lever to balance benefits and risks. Under an identical total N input (270 kg ha^−1^), increasing the topdressing proportion (N1) significantly improved grain yield (12.2% higher than CK), whereas shifting more N to the basal application (N2) tended to drive the system toward a high-risk, low-return state. Overall, these results corroborate previous findings that split N application and delaying N supply can enhance N use efficiency and improve yield components [31,32], and they are also consistent with the view that excessive early-season N availability can promote canopy overgrowth, thereby increasing lodging susceptibility without necessarily translating into yield gains [30,33].

From an operational and economic perspective, optimizing the basal-to-topdressing N ratio is a low-cost and readily implementable strategy. Under unchanged total N input and without increasing the number of fertilizer applications (still basal application plus topdressing at jointing), simply reallocating N between the two splits achieved higher grain yield and lower lodging risk. This implies potential economic gains through yield improvement and loss reduction without additional fertilizer input; meanwhile, reduced lodging risk can also lower operational difficulty and losses during late-season management and harvesting [34,35]. Compared with practices that require additional inputs or extra field operations, this strategy offers greater scalability and reproducibility for on-farm adoption.

Notably, the recommended basal-to-topdressing ratio is likely context-dependent and may vary with water regime and management intensity; for example, some studies under micro-sprinkler irrigation have reported a 5:5 split to be more favorable for increasing yield or resource-use efficiency [10,32]. In contrast, our N1 treatment delivered a more advantageous overall outcome under lodging-prone conditions. In the 2022–2023 season, when lodging occurred, mean yield declined by 18.4% relative to the preceding season, and lodging events were concentrated during the milk stage following windy and rainy weather, indicating that lodging became a major constraint on yield realization in this year type. Therefore, the superiority of N1 was not merely reflected by achieving the highest yield, but rather by providing a more robust balance between yield gain and risk mitigation under lodging-sensitive conditions, making it a more broadly applicable N management strategy.

### 3.2. Coupled Regulation of I2 Traits by the Basal-to-Topdressing N Ratio and Cultivar Characteristics

A large body of evidence suggests that PH and CGH can modulate lodging risk by altering the bending moment [36,37,38]; however, these traits are not determinative. Rather, lodging resistance is ultimately governed by the structural quality and tissue-level attributes of the key basal internodes [12,39]. The cultivar contrasts observed here support this framework: ML maintained consistently stronger I2 mechanical performance across growth stages (overall higher CLRI and BS) and did not lodge under any basal-to-topdressing N ratio, whereas JH exhibited the weakest overall performance but still did not lodge under N1, indicating that cultivar background modulates the marginal effectiveness of management rather than acting as a sole determinant. This is consistent with genetic perspectives emphasizing that anatomical architecture and cell-wall reinforcement capacity define the inherent lodging-resistance potential of wheat cultivars [40,41].

Furthermore, the differences among the basal-to-topdressing N ratio treatments provide additional management-level evidence supporting this notion. The N1 treatment increased PH and CGH to some extent, which would theoretically elevate the bending moment by lengthening the lever arm and thereby increase lodging susceptibility [38,42]. However, N1 more strongly promoted structural and tissue-level reinforcement of I2, resulting in a larger improvement in load-bearing capacity against stem breakage. Because CLRI is essentially an integrative measure of strength relative to the lever arm, its net change can be approximately decomposed as the difference between the proportional gain in breaking strength and the proportional increase [43,44]. Accordingly, under N1, even when PH and CGH increased, CLRI still rose as long as BS increased proportionally more. Together, these results indicate that improving lodging resistance hinges on enhancing multi-scale, coordinated reinforcement of I2 such that the structural gains are sufficient to offset the bending-moment risk associated with greater plant height.

More importantly, we found that the effects of the basal-to-topdressing N ratio on I2 traits were broadly consistent across cultivars. In all three cultivars, N1 shifted I2 traits in a more favorable direction, whereas CK and N2 more readily exposed risk in cultivars with insufficient structural quality (notably JH). Nevertheless, we do not exclude the possibility that, for lodging-tolerant semi-dwarf cultivars such as ML, a moderate reduction in topdressing proportion could be considered, potentially increasing spike number without compromising lodging resistance [10,30]. Collectively, these findings indicate a coupled regulation between N-splitting strategy and cultivar structural attributes, suggesting that optimizing the basal-to-topdressing ratio in alignment with cultivar architecture may help achieve the dual objective of high-yield plant types while maintaining strong lodging resistance.

### 3.3. Mechanistic Interpretation of Multi-Scale Trait Coordination in I2 Reinforcement Within the Lodging-Sensitive Window

Mechanistically, previous studies generally report that higher lignin and cellulose contents are positively associated with improved culm strength and enhanced lodging resistance [24,45,46], whereas other work has highlighted potential antagonism or trade-offs between cellulose and lignin due to cross-linking or structural constraints [47,48]. Our multi-scale dataset provides an integrated perspective that helps reconcile these seemingly divergent views. At the anatomical level, increasing the topdressing proportion (N1) consistently enhanced I2 vascular bundle development, reflected by higher numbers and areas of both small and large vascular bundles and greater mechanical tissue thickness, in line with the notion that vascular-bundle architecture and mechanical tissues underpin stem load-bearing capacity [17].

At the compositional level, SSC increased after anthesis and peaked at the milk stage, SC progressively declined, whereas LC and CC accumulated continuously, indicating that the period around the milk stage represents a key window for carbon reallocation and cell-wall reinforcement. Under N1, LC was higher than under CK and N2, and LC and CC could increase concurrently in ML, supporting the prevailing view that these wall components jointly contribute to culm strength [8,49]. Importantly, under an appropriate N allocation strategy and a favorable genetic background, structural wall materials may accumulate synergistically [50], thereby maximizing strength gains—potentially explaining why no antagonistic pattern was evident in our study. By contrast, although N2 showed advantages in I2 nitrogen accumulation and SIDW, it was associated with lower CLRI and weaker wall-material accumulation, suggesting that N accumulation or biomass gain cannot substitute for structural-quality contributions. This may be related to a mechanism whereby excessive N alters assimilate partitioning, thereby reducing the supply of structural assimilates to the culm and leading to insufficient deposition of wall materials [8,45]. However, we did not quantitatively measure the relevant indicators; therefore, this explanation should be regarded as a mechanistically informed inference supported by the observed compositional patterns.

Furthermore, correlation and structural equation modeling jointly connected the above multi-scale evidence into a coherent, interpretable pathway. Yield was positively correlated with CLRI, and CLRI was positively associated with TDI, TCWT, MT, and key compositional traits, but negatively associated with height-related traits such as PH and CGH, highlighting the need for dynamic balancing between yield potential (morphological expansion) and lodging stability (structural reinforcement) through N management. The SEM-supported “composition–structure–lodging resistance–yield” cascade emphasizes that CLRI acts as a pivotal mediator linking I2 reinforcement to yield gains. Under the present experimental conditions, the superiority of N1 can therefore be attributed to its concurrent enhancement of wall-material deposition, anatomical reinforcement, and geometric/structural traits, which together elevate the mechanical threshold during the milk-stage lodging window and reduce lodging probability.

### 3.4. Limitations and Perspectives for Application

Overall, our findings support the view that increasing the topdressing proportion is a key management lever for simultaneously improving lodging resistance and sustaining yield under high-yield conditions. However, this study covered only two growing seasons, with lodging events occurring predominantly in 2022–2023 and within a relatively narrow developmental window; therefore, the robustness and boundary of applicability of the N1 strategy still need to be further validated across a wider range of year types. It should be emphasized that, under a well-characterized soil background and documented meteorological conditions at the experimental site, we set the total N rate at a locally representative input level for high-yield production and maintained identical total N supply and irrigation regimes across treatments, varying only the basal-to-topdressing N ratio to minimize confounding from differences in N rate and water status. Accordingly, conclusions regarding the split ratio should be interpreted as a recommended basis under soil–management settings comparable to those of this experiment, rather than as a universally applicable rule across contrasting soils and climates. Therefore, the basal-to-topdressing N ratio should not be considered fixed; in practice, I2 structural quality should be treated as the primary constraint, and cultivar-specific optimization of N split ratios should be prioritized to provide more actionable targets for lodging-resistance breeding and precision crop management.

In addition, lodging is strongly influenced by interactions between water processes and N management. Although we implemented micro-sprinkler irrigation as a “small but frequent” regime at jointing, booting/anthesis, and grain filling and kept the irrigation schedule identical across plots, the matching between irrigation regimes and the timing/ratio of topdressed N may still affect lodging risk under different rainfall year types (drought versus high rainfall) and contrasting soil water-supply capacities. Future work should validate these findings across multiple locations and years under multi-environment conditions, encompassing contrasting climatic scenarios such as drought and high rainfall, and calibrate both total N input and split strategies using indices of soil N supply capacity (e.g., soil Nmin testing) [51]. In parallel, comparisons among alternative topdressing timings (e.g., adding booting-stage topdressing on top of jointing) and split frequencies would help improve the adaptability of N-splitting strategies under varying soils and climate trends. Meanwhile, lodging is also modulated by canopy aerodynamic drag, root anchorage, and soil water status [52,53]; future studies could integrate key processes including aboveground loading and belowground support into a unified framework to strengthen mechanistic interpretation and improve predictive capability under extreme weather conditions.

## 4. Materials and Methods

### 4.1. Experimental Site

**Figure 8:**
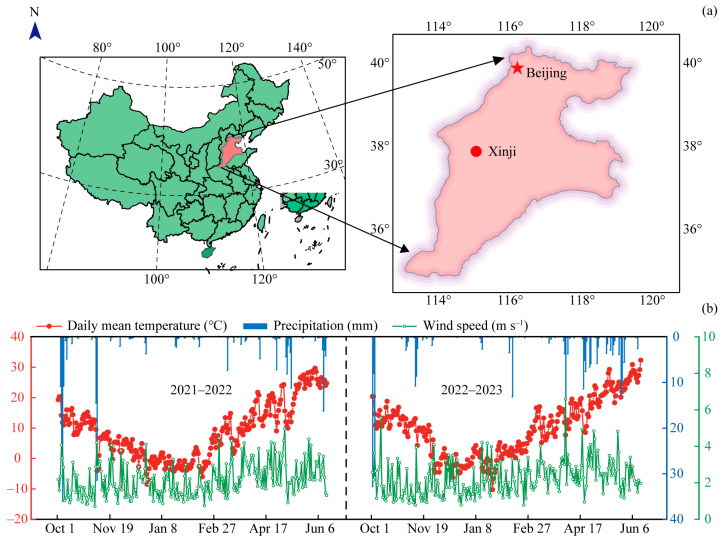
Location of the experimental site (**a**), and daily mean temperature, precipitation, and wind speed at 2 m height at the experimental station during the winter wheat growing seasons from 2021 to 2023 (**b**).

Field experiment was conducted at the Malan Experimental Station, Xinji City, Hebei Province, China (37°59′29″ N, 115°12′01″ E) during the 2021−2023 winter wheat growing seasons (Figure 8a). The site is located in a semi-humid, temperate continental monsoon climate zone at an elevation of 37 m above sea level, with a cinnamon loam soil. The topsoil had a bulk density of 1.50 g cm^−3^, soil organic matter of 20.70 g kg^−1^, total nitrogen of 1.09 g kg^−1^, alkali-hydrolysable nitrogen of 65.11 mg kg^−1^, available phosphorus of 20.40 mg kg^−1^, and available potassium of 106.02 mg kg^−1^. The precise and dependable meteorological data of this region were sourced from the China Meteorological Data Network (http://data.cma.cn/). The changes in daily mean temperature, precipitation, and wind speed at 2 m height during the experiment are shown (Figure 8b).

### 4.2. Experimental Design and Field Management

A two-factor randomized block design, with cultivar as factor I and the basal-to-topdressing N ratio as factor II, was used in the experiment; factor I was used at three cultivars [Malan No.1 (ML), Jimai 22 (JM), and Jinhe 9123 (JH)], while factor II was used at three split-ratio levels [5:5 (CK), 3:7 (N1), and 7:3 (N2)]. Each treatment was replicated three times, and each plot covered 36 m^2^. These cultivars are locally representative and differ in plant stature and culm-related traits, thereby providing contrasting lodging-resistance backgrounds (Table 2). To facilitate comparison of the effects of different basal-to-topdressing N ratios under the same total N supply and to minimize confounding arising from differences in total N rate, the total N rate was uniformly set to 270 kg ha^−1^ (N rate was determined by local extension agents based on a target yield equal to 1.1 times the average yield of the previous five years [54]), which represents a typical N input level under local high-yield production practice at the experimental site. Nitrogen fertilizer was applied in two splits as follows: basal N before sowing (incorporated into the soil during rotary tillage) and topdressed N at jointing (applied with irrigation). Micro-sprinkler irrigation was used in spring at jointing, booting/anthesis, and grain filling, with irrigation amounts of 60, 45, and 30 mm, respectively, and the irrigation schedule was identical across plots. All other field management practices followed the local high-yield production protocol.

### 4.3. Sampling and Measurement

#### 4.3.1. Lodging Assessment

Lodging was evaluated according to the Chinese national standard GB17317-1998 and classified into the following four grades based on the angle between the culm and the ground surface [55]: grade 0 (>75°), grade I (60–75°), grade II (30–60°), and grade III (<30°). The lodging rate was calculated as follows [56]:

(1) $$\mathrm{Lodging}\;\mathrm{rate}\;\left(\%\right)\;=\;\frac{A_{\mathrm{lodged}}}{A_{\mathrm{plot}}}\;\times \;100$$

where A_lodged_ is the actual lodged area in a plot, and A_plot_ is the total plot area.

#### 4.3.2. Stem Morphological Measurements

At anthesis, 30 main stems were randomly selected from each plot. PH was measured as the distance from the stem base to the tip of the spike, and the length of I2 was also recorded. At anthesis, milk stage, and dough stage, 30 main stems were randomly sampled from each plot, and the distance (cm) from the stem base to the balancing point of the intact culm (including spike, leaves, and sheaths) was measured [57]. The mean value was taken as the CGH. After removing the leaf sheaths, the inner and outer diameters and the length of I2 were measured. The outer diameter was defined as the TDI, and TCWT was calculated as

(2) $$\mathrm{TCWT}=\frac{D_{\mathrm{outer}}-D_{\mathrm{inner}}}{2}$$

where D_outer_ and D_inner_ are the outer and inner diameters of I2, respectively [58].

Finally, the entire I2 segment was excised and oven-treated at 105 °C for 40 min to deactivate enzymes, followed by drying at 70 °C to constant weight. The dried I2 was weighed, and TFD was calculated as

(3) $$\mathrm{TFD}=\frac{W_{\mathrm{dry}}}{L}$$

where W_dry_ is the dry weight of I2 and L is the I2 length [59].

#### 4.3.3. Stem Mechanical Measurements

At anthesis, milk stage, and dough stage, 30 main stems were randomly sampled from each plot. The breaking strength (BS) of the I2 was determined using a stem strength tester (YYD-1, Zhejiang Top Cloud-Agri Technology Co., Ltd., Hangzhou, China). The CLRI was then calculated as [15]

(4) $$\mathrm{CLRI}=\frac{\mathrm{BS}}{\mathrm{CGH}}$$

#### 4.3.4. Determination of Culm Constituents

At anthesis, milk stage, and dough stage, 30 plants with uniform growth were selected from each plot. After removing leaf sheaths, the I2 was excised, oven-dried, and used for culm constituent analysis. The assay procedures were as follows.

Cellulose content was determined according to the method of Hussain et al. [60]. Dried samples were ground to a fine powder using a Mixer Mill MM 400 (Retsch GmbH, Haan, Germany) and passed through an 80-mesh sieve. An aliquot of 0.15 g powder was sequentially washed with 80% ethanol and acetone, followed by centrifugation and removal of the supernatant. The residue was incubated with 1 mL dimethyl sulfoxide (DMSO) at 25 °C for 15 h, centrifuged at 4000× *g*, the supernatant was discarded, and the remaining material was oven-dried to constant weight. A subsample (0.005 g) was mixed with 0.5 mL distilled water by vortexing, after which 750 μL concentrated sulfuric acid was added slowly and the mixture was incubated in an ice-water bath for 30 min. The mixture was centrifuged at 8000× *g* for 10 min at 4 °C, and the supernatant was diluted 20-fold with distilled water. A 300 μL aliquot of the diluted solution was then reacted with 70 μL ethyl anthrone acetate and 630 μL concentrated sulfuric acid. After incubation in a 95 °C water bath for 10 min, absorbance was measured at 620 nm, and CC was calculated as

(5) $$\mathrm{CC}=4.76\;\times \;\frac{\Delta A+0.00043}{W}$$

where ΔA is the absorbance at 620 nm and W is the sample weight (g).

Lignin content was determined following the method of Cheng et al. [61]. Dried samples were ground to a fine powder and passed through an 80-mesh sieve. A 0.10 g subsample was mixed with 2.5 mL of 25% acetyl bromide solution (glacial acetic acid: acetyl bromide = 4:1, *v*/*v*) and 1.0 mL perchloric acid, and the mixture was thoroughly vortexed. The tubes were incubated in a water bath at 80 °C for 40 min with intermittent shaking every 10 min; a reagent blank without sample was included. After cooling to room temperature, a 0.5 mL aliquot was neutralized with 2 mol L^−1^ NaOH and hydroxylamine hydrochloride. Subsequently, 10 μL of the neutralized solution was added to glacial acetic acid, mixed well, and the absorbance was measured at 280 nm using a quartz cuvette. LC was calculated as

(6) $$\mathrm{LC}=0.0735\;\times \;\frac{\Delta A\;-\;0.0068}{W}\;\times \;T$$

where ΔA is the absorbance at 280 nm, W is the sample weight (g), and T is the dilution factor.

For nitrogen determination, dried I2 samples were pulverized using a high-speed grinder, passed through a 100-mesh sieve, and digested using the H_2_SO_4_–H_2_O_2_ digestion method to prepare the chromogenic solution; nitrogen concentration was then quantified with a SmartChem 200 discrete analyzer (AMS Alliance, Guidonia, Italy). Soluble sugar content and starch content were determined using the anthrone colorimetric method.

#### 4.3.5. Grain Yield and Yield Components

In each plot, three representative 1 m^2^ quadrats were selected to determine spike number. Thirty spikes were randomly sampled to calculate kernels per spike. After harvest, grain yield per unit area and thousand-kernel weight were determined on a 13% standard moisture basis.

#### 4.3.6. Association of Key Traits and Mechanistic Pathway Analysis of Lodging Resistance

Based on observations at the MS, Pearson’s correlation analyses were performed among yield, CLRI, BS, and the remaining candidate traits, and the significance of correlation coefficients was tested (*p* < 0.05). To quantify the overall associations between the candidate traits and each of yield, CLRI, and BS, all variables were standardized (z-scores) prior to analysis, Euclidean distance matrices were constructed, and Mantel’s r and its significance were assessed using permutation tests (≥999 permutations). Subsequently, a SEM was specified according to the correlation and Mantel results in conjunction with biologically plausible hypotheses. Candidate traits were integrated into three latent modules—morphological traits, anatomical traits, and stem chemical composition—with CLRI specified as a key mediator and yield as the terminal outcome, and standardized path coefficients were estimated. Overall model fit was evaluated using the χ^2^ test and commonly used fit indices, and non-significant paths were pruned while maintaining biological interpretability. In addition, because CLRI is a derived index, BS was not explicitly included in the SEM to minimize mathematical coupling, whereas the CGH was retained as an essential component of the morphological phenotype.

#### 4.3.7. Statistical Analysis

Routine analyses of variance were performed in SPSS 26.0 (SPSS Inc., Chicago, IL, USA). For datasets meeting the assumptions of ANOVA, treatment means were compared using Tukey’s HSD test at *p* < 0.05 when the ANOVA F-test was significant; different lowercase letters indicate significant differences among treatments. Correlation analyses were conducted in SPSS, whereas Mantel tests and SEM were implemented in R (R Foundation for Statistical Computing, Vienna, Austria). Figures were generated and formatted using Origin 2022 (OriginLab, Northampton, MA, USA) and Adobe Illustrator 2021 (Adobe Inc., San Jose, CA, USA).

## 5. Conclusions

This study integrates multiple indicators of morphological structure, anatomical structure, composition, and mechanical characteristics, focusing on the basal load-bearing core of wheat—I2. A clear composition–structure–lodging resistance–yield pathway was identified, identifying the CLRI as the key mediator linking I2 trait reinforcement and yield realization. Accordingly, a recommended nitrogen basal-to-topdressing ratio strategy (N1) was identified under our experimental conditions, balancing both yield and lodging resistance. This strategy is also operationally feasible for on-farm adoption and offers potential economic benefits. The findings provide actionable nitrogen distribution principles and risk management strategies for winter wheat production in NCP, while offering a direct breeding target based on I2 structural quality for lodging resistance. Future research could validate the robustness and applicability of the N1 strategy across more year types, incorporating additional lodging impact indicators to further refine nitrogen management strategies.

## Figures and Tables

**Table 1:** Yield and yield components of different treatments in 2021–2022 and 2022–2023 growing seasons.

Year	Treatment		Spike Number	Grain Number per Spike	Thousand Grain Weight	Yield
			(10^4^ ha^−1^)		(g)	(kg ha^−1^)
2021–2022	ML	CK	686.4 ± 12.2 b	31.6 ± 0.8 abc	47.4 ± 1.1 ab	10,074.4 ± 40.3 bc
		N1	752.3 ± 6.3 a	32.7 ± 1.0 ab	48.6 ± 0.8 a	11,242.7 ± 300.0 a
		N2	672.4 ± 2.5 bcd	31.1 ± 0.7 bc	44.9 ± 0.5 cd	9255.2 ± 76.2 d
	JH	CK	653.0 ± 15.7 cd	30.7 ± 0.9 c	47.7 ± 0.6 ab	9415.8 ± 35.6 d
		N1	644.8 ± 21.1 d	32.7 ± 0.1 ab	49.2 ± 0.5 a	10,132.5 ± 25.7 bc
		N2	642.5 ± 3.2 d	29.9 ± 0.3 c	46.4 ± 0.5 bc	9087.6 ± 42.7 d
	JM	CK	696.2 ± 6.7 b	32.6 ± 0.6 ab	44.0 ± 0.3 d	9945.0 ± 86.1 c
		N1	690.5 ± 11.2 b	33.0 ± 0.3 a	47.7 ± 1.0 cd	10,329.7 ± 107.0 b
		N2	683.3 ± 2.0 bc	32.9 ± 0.4 a	41.2 ± 0.6 e	9128.0 ± 73.0 d
2022–2023	ML	CK	532.5 ± 10.4 b	36.3 ± 0.2 ab	49.6 ± 1.0 ab	8708.6 ± 51.0 c
		N1	571.9 ± 8.3 a	37.6 ± 0.4 a	52.2 ± 0.9 a	10,768.5 ± 582.8 a
		N2	463.2 ± 6.7 d	32.8 ± 1.0 c	42.5 ± 0.6 e	6570.9 ± 79.6 e
	JH	CK	464.8 ± 12.6 d	34.3 ± 0.5 bc	45.5 ± 1.1 cde	7197.9 ± 156.8 d
		N1	508.6 ± 4.5 c	36.5 ± 0.6 ab	46.4 ± 0.3 bcd	8705.1 ± 90.2 c
		N2	457.5 ± 4.5 d	30.0 ± 1.1 d	43.2 ± 2.0 de	5907.5 ± 46.2 f
	JM	CK	535.7 ± 4.0 b	34.7 ± 0.9 bc	46.2 ± 1.1 cd	8678.5 ± 52.8 c
		N1	553.8 ± 9.0 ab	35.3 ± 0.9 ab	47.4 ± 1.5 bc	9449.7 ± 94.6 b
		N2	435.4 ± 2.9 e	32.5 ± 1.2 c	44.7 ± 0.8 cde	6330.4 ± 24.1 ef
ANOVA	Year (Y)		***	***	**	***
	Variety (V)		***	***	***	***
	Nitrogen (N)		***	***	***	***
	Y × V		**	***	***	***
	Y × N		***	***	***	***
	V × N		***	***	***	***
	Y × V × N		***	ns	***	*

Y, year; V, variety; N, nitrogen. Different lowercase letters within a column indicate significant differences among treatments within the same year at *p* < 0.05 (*n* = 3). * indicates significance at the level of 0.05, ** indicates significance at the level of 0.01, *** indicates significance at the level of 0.001, ns indicates not significant at the level of 0.05.

**Table 2:** Comparison of agronomic descriptors of the three winter wheat cultivars used in this study.

Cultivar	Plant Height (cm)	Plant Type	Stem	Tillering Capacity	Locally Dominant Cultivar (NCP)	Lodging Resistance
ML	~68.5	V-shaped	Thick, rigid	Medium–high	Yes	Strong
JH	~75.0	Compact	Relatively thin	Strong	Yes	Poor
JM	~72.0	Compact	Relatively thick, elastic	Medium	Yes	Moderate

## Data Availability

Data are contained within the article.

## Abbreviations

The following abbreviations are used in this manuscript:

NCP	North China Plain
I2	Second Basal Internode
N	Nitrogen
CLRI	Culm Lodging Resistance Index
SEM	Structural Equation Modeling
TDI	Stem Diameter of the Second Basal Internode
TCWT	Culm Wall Thickness
TFD	Filling Degree
SVN	Number of Small Vascular Bundles
BVN	Number of Large Vascular Bundles
ASV	Area of Small Vascular Bundles
ABV	Area of Large Vascular Bundles
MT	Mechanical Tissue Thickness
SSC	Soluble Sugar Content
SC	Starch Content
LC	Lignin Content
CC	Cellulose Content
NC	Nitrogen Content
BS	Breaking Strength
PH	Plant Height
CGH	Center of Gravity Height
CK	Control Treatment
N1	Nitrogen Treatment with 3:7 Split Ratio
N2	Nitrogen Treatment with 7:3 Split Ratio
